# Long-term monitoring of the fish community in the Minho Estuary (NW Iberian Peninsula)

**DOI:** 10.3897/BDJ.12.e112217

**Published:** 2024-03-08

**Authors:** Allan T. Souza, Martina I Ilarri, Ester Dias, Mário J Araújo, António Roleira, Ana Catarina Braga, Ana Rita Carvalho, Micaela Mota, Maria Helena Correia, Ana Lages, Ana Moura, Carlos Antunes

**Affiliations:** 1 Institute for Atmospheric and Earth System Research INAR, Forest Sciences, Faculty of Agriculture and Forestry, P.O. Box 27, 00014 University of Helsinki, Helsinki, Finland Institute for Atmospheric and Earth System Research INAR, Forest Sciences, Faculty of Agriculture and Forestry, P.O. Box 27, 00014 University of Helsinki Helsinki Finland; 2 Interdisciplinary Centre of Marine and Environmental Research (CIIMAR/CIMAR), University of Porto, Novo Edifício do Terminal de Cruzeiros do Porto de Leixões, Av. General Norton de Matos, s/n, 4450-208, Matosinhos, Portugal Interdisciplinary Centre of Marine and Environmental Research (CIIMAR/CIMAR), University of Porto, Novo Edifício do Terminal de Cruzeiros do Porto de Leixões, Av. General Norton de Matos, s/n, 4450-208 Matosinhos Portugal; 3 S2AQUA—Collaborative Laboratory, Association for a Sustainable and Smart Aquaculture, Av. Parque Natural da Ria Formosa s/n, 8700-194, Olhão, Portugal S2AQUA—Collaborative Laboratory, Association for a Sustainable and Smart Aquaculture, Av. Parque Natural da Ria Formosa s/n, 8700-194 Olhão Portugal; 4 Aquamuseu do Rio Minho, Parque do Castelinho, 4920-290, Vila Nova de Cerveira, Portugal Aquamuseu do Rio Minho, Parque do Castelinho, 4920-290 Vila Nova de Cerveira Portugal

**Keywords:** abundance, biodiversity, ecology, fyke nets, ichthyology

## Abstract

**Background:**

The paper presents an extensive fish sampling dataset spanning a long-term period from 2010 to 2019. The data were collected in Lenta Marina, an upstream area in the Minho Estuary of the NW Iberian Peninsula, which belongs to a LTSER (Long-Term Socio-Ecological Research) platform. To capture fish, fyke nets were utilised as the sampling method and deployed at Lenta Marina. This dataset offers valuable insights into the abundance of each collected taxa recorded over time.

**New information:**

The dataset reports a comprehensive compilation of data on the abundance of fish species observed in the area during the sampling period (includes zeroes when a given taxonomic entity was absent in a given sampling event). It provides a detailed record of the abundances of the fish community through time in a frequent sampling regime (on average, sampling was done every 6 days). The dataset shows that the amount of fish from invasive taxa exceeds the count of fish from native taxa in the Minho Estuary.

## Introduction

Estuarine ecosystems play an important role in maintaining fish biodiversity. They are highly biologically productive ecosystems that provide important feeding, spawning, refuge and nursery habitats for various fish species at different stages of development (Cabral et al. 2007, Guerreiro et al. 2021). Despite their high ecological and socio-economic value, estuaries are currently stressed by a variety of anthropogenic activities, including nutrient over-enrichment (Nixon 2009), habitat loss (Stamp et al. 2022), overexploitation of natural resources (Vasconcelos et al. 2007), the introduction of invasive species (Levin and Crooks 2011), altered freshwater inflows (Palmer et al. 2011), pollution (Gao et al. 2019) and climate change (Ilarri et al. 2022, Souza et al. 2023). It is, therefore, important to establish long-term monitoring programmes that provide an accurate overview of the state of these ecosystems and how different species populations respond to these different pressures (in terms of abundance and population composition) over the years.

For over a decade, the ichthyofauna within the Minho Estuary, located in the north-western region of the Iberian Peninsula, has been subject to consistent monitoring (Souza et al. 2023). The Minho River has been recognised as an EU Natura 2000 site and its estuary has obtained Special Protection Area status under the EU Birds Directive (Directive 2009/147/EC). This area is home to significant bird, macroinvertebrate and fish populations, including various migratory fish species (Mota et al. 2014). However, the ecological condition of the Minho Estuary has experienced a decline in recent decades, attributed to a combination of factors such as overfishing, pollution, the invasion of alien species and fluctuations in weather conditions (Sousa et al. 2011, Mota and Antunes 2012, Ilarri et al. 2014, Beiras 2016, Ilarri et al. 2022, Souza et al. 2023).

The objective of this study was to document the sampling effort and the abundance and composition of the fish community (allowing the calculation of the CPUE - Catch Per Unit Effort) inhabiting the Minho Estuary between 2010 and 2019. The primary goal was to record the temporal changes that have occurred within the fish community through time.

## Project description

### Study area description

**Figure 1.**
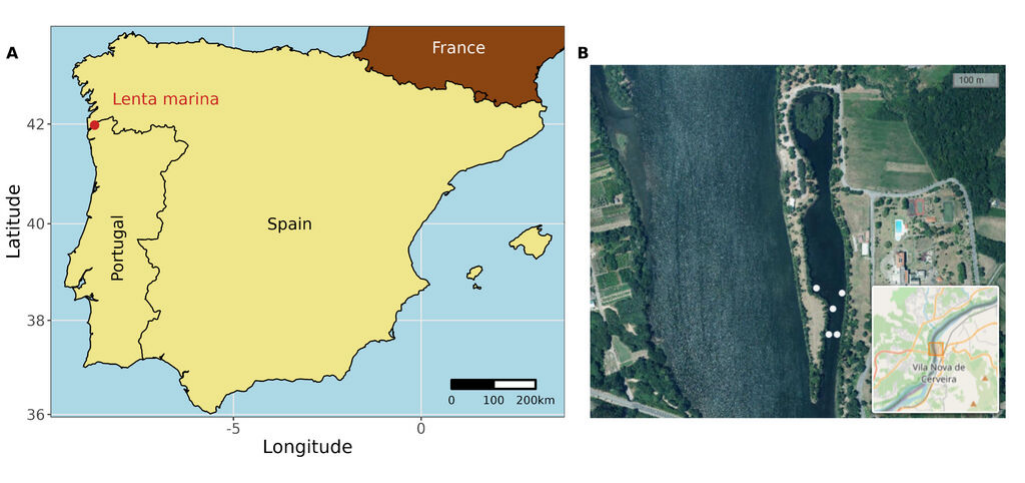
Spatial representation of the study area and sampling locations in the Minho Estuary. A) A map of the Iberian Peninsula, with an indication of the sampling location within the Minho Estuary. B) A zoomed-in view of the study area, highlighting the representative deployment locations of the fyke nets (white circles) inside the Lenta Marina.

The research was carried out in the LTSER-ESTUARIES - Portugal (https://deims.org/664177a4-a21a-4f59-9601-00909e275868), more especially at the Lenta Marina (41°57'15.9"N 8°44'42.5"W), a small (660 × 80 m), semi-enclosed bay situated 14.5 km upstream in the Minho Estuary, which was chosen as a representative area of the estuary (Figs 1, 4). Lenta Marina occupies a location within an estuarine zone characterised by slight salinity fluctuations, with freshwater prevailing in accordance with the patterns of discharge of the river. In summer, Lenta Marina experiences minor saline intrusions, leading to relatively low salinity levels in the area. Specifically, salinity can range from 0 - 2 psu during late summer and drought conditions (Dias et al. 2016, Dias et al. 2020). The Minho Estuary is classified as a mesotidal estuary, characterised by an average depth of 2.6 m and reaching a maximum depth of 26 m (Alves 1996, Antunes et al. 2011).

### Funding

This research was supported by national funds through FCT – Foundation for Science and Technology within the scope of UIDB/04423/2020 and UIDP/04423/2020. This work was partly carried out in the framework of the Migra Miño Minho project "Protection and conservation of migratory fish in the conservation of migratory fish in the international stretch of the River Minho and its tributaries", a project co-financed by the European Regional Development Fund (ERDF) through the Interreg V-A Programme, Spain through the Interreg V-A Programme, Spain-Portugal (POCTEP), 2014-2020

## Sampling methods

### Sampling description

**Figure 2.**
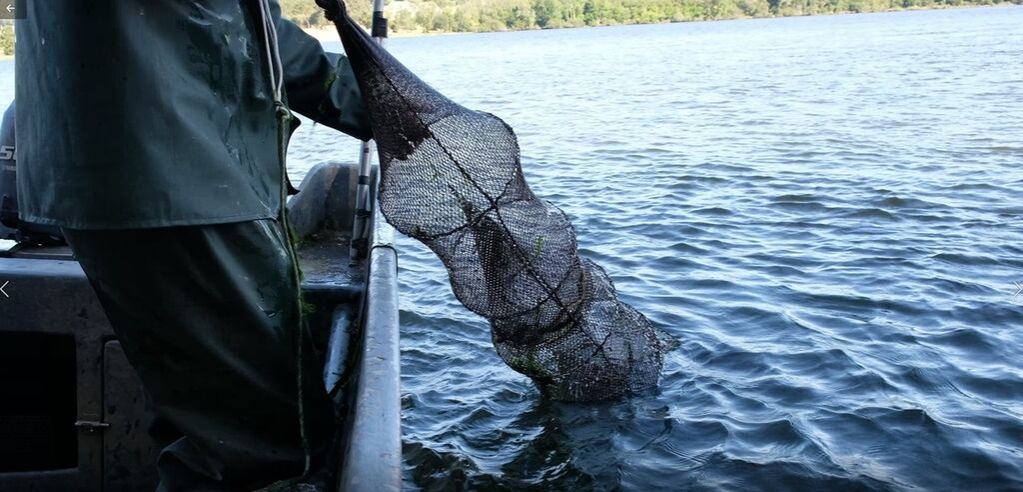
A photograph showing the removal of a fyke net from the water in the Minho Estuary (Photo by Carlos Antunes).

**Figure 3.**
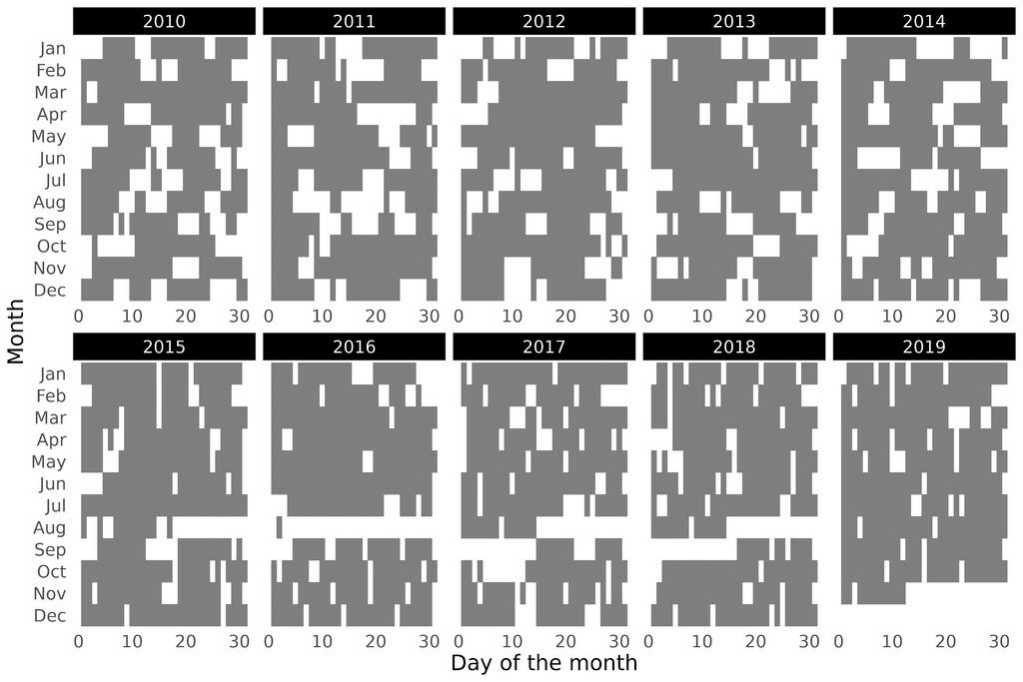
Days when samples were collected in the study area (Lenta Marina) in the Minho Estuary (i.e. days when fyke net(s) were underwater). Grey = fyke net(s) deployed; white = no fyke net deployed.

**Figure 4.**
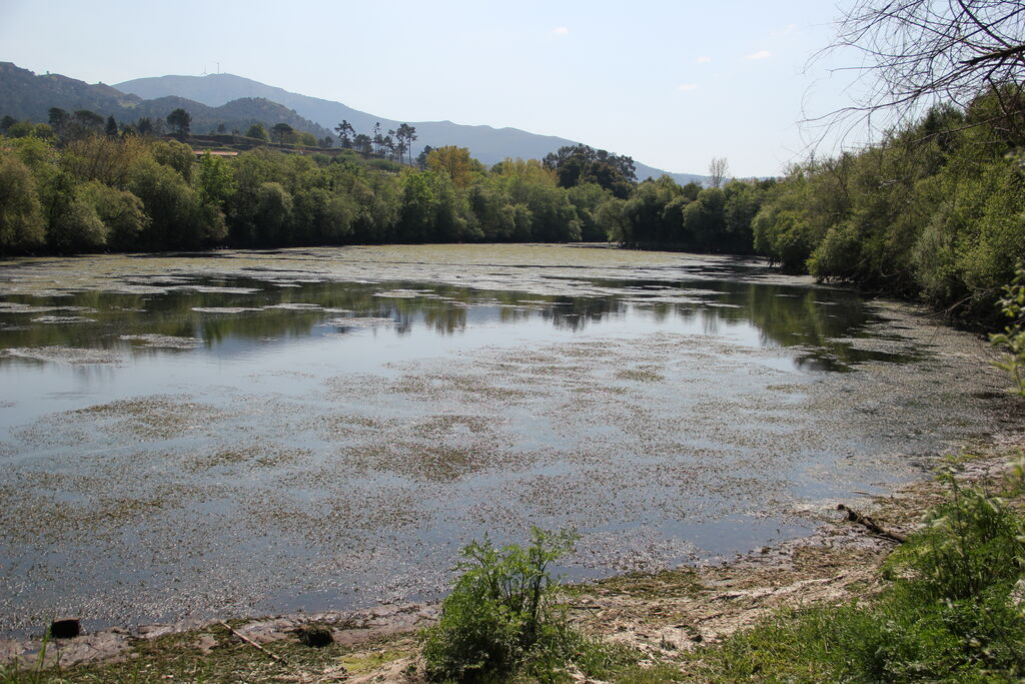
A photograph showing the view of the study area (Lenta Marina) in the Minho Estuary. The image shows the semi-enclosed bay, characterised by the presence of near-shore vegetation and the calm water surface where the fish sampling took place (Photo by Ronaldo Sousa).

**Figure 5.**
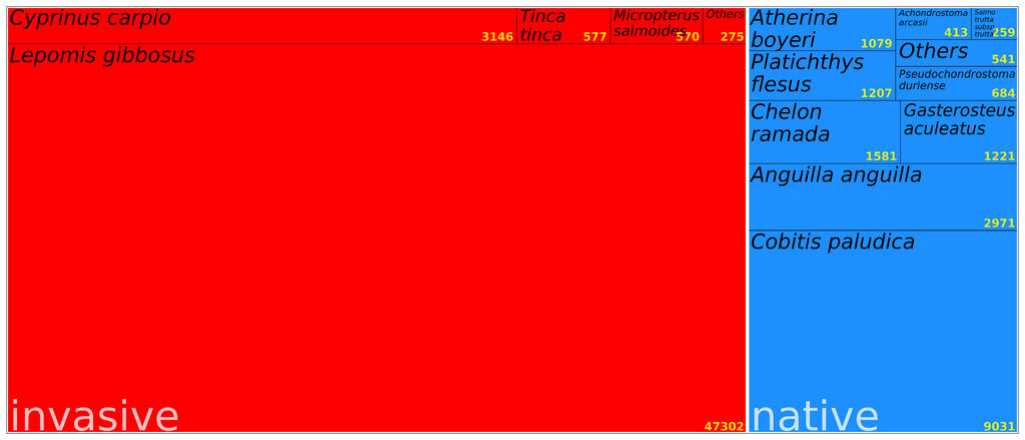
Treemap showing the total number of individuals of the most representative species during the long-term monitoring. The area of each rectangle is proportional to the total abundance of the species in all samples collected and the yellow numbers in the bottom right corner indicate the total abundance per species. Red background colour refers to the invasive species, with the category others corresponding to the least abundant species in the group, in this case, representative of two species: *Gobiolozanoi* (n = 255) and *Carassiusauratus* (n = 20). Blue background colour refers to the native species, with the category others corresponding to the least abundant species in the group, in this case, it is representative of eight species: *Mugilcephalus* (n = 167), Salmotruttasubsp.fario (n = 162), *Dicentrarchuslabrax* (n = 100), *Chelonauratus* (n = 51), *Chelonlabrosus* (n=28), *Squaliuscarolitertii* (n = 20), *Petromyzonmarinus* (n = 12) and *Alosa* (n = 1).

Fish samples were collected from January 2010 to November 2019. Fyke nets with a mesh size of 10 mm, a length of 7 m, a mouth diameter of 0.7 m and a central wing of 3.5 m were used to collect the fish (Fig. 2). These nets were consistently set in the morning and left submerged for 5.5 ± 2.6 days (average ± SD) (Fig. 3). To ensure even spatial coverage, the fyke nets were always set parallel to the shore and distributed close to the bay mouth at fixed locations (Fig. 1). Although the number of fyke nets used per sampling date varied due to technical issues (e.g. lost or damaged fyke nets), up to five fyke nets were employed per sampling date. After retrieving of the fyke nets, all captured individuals were identified (following Froese and Pauly (2023) and Martins and Carneiro (2018)) and counted (Fig. 5). During the entire study period, a total of 3,029 samples (i.e. individual fyke nets) were collected.

Permission to collect fish within the study area was obtained in correspondence with the Portuguese Navy.

### Quality control

It is important to note that fyke netting is a passive gear and, therefore, considered a semi-quantitative method, which can be influenced by gear catchability and saturation, fish activity and behaviour, predation inside fyke nets, as well as environmental conditions (e.g. water temperature and transparecy) (Hubert and Fabrizio 2007). The use of this dataset needs to account for these known issues.

## Geographic coverage

### Description

This study was carried out in Lenta Marina in the Minho Estuary in the north-west of the Iberian Peninsula.

### Coordinates

41°57'15.9'' and 41°57'15.9'' Latitude; 8°44'42.5'' and 8°44'42.5'' Longitude.

## Taxonomic coverage

### Description

The dataset comprised the records of 70,857 individuals belonging to 20 species, two subspecies and one genus (which has not been identified to the species level) taxa of fish from 12 families. The taxonomic list was defined, based on the presence of a taxonomic group in the entire dataset and the presence or absence of each taxonomic group in a eventID was recorded at occurrenceStatus. The taxon identification numbers (acceptedNameUsageID) were based on GBIF Backbone Taxonomy (GBIF Secretariat 2022), whereas the common names of the species (vernacularName) were based on FishBase (Froese and Pauly 2023).

### Taxa included

**Table:** 

Rank	Scientific Name	Common Name
species	*Petromyzonmarinus* Linnaeus, 1758	Sea lamprey
species	*Anguillaanguilla* (Linnaeus, 1758)	European eel
genus	*Alosa* Linck, 1790	Allis and twaite shads
species	*Cobitispaludica* (de Buen, 1930)	Iberian loach
species	*Achondrostomaarcasii* (Steindachner, 1866)	Panjorca
species	*Pseudochondrostomaduriense* (Coelho, 1985)	Douro nase
species	*Squaliuscarolitertii* (Doadrio, 1988)	Iberian chub
subspecies	Salmotruttasubsp.fario Linnaeus, 1758	Brown trout
subspecies	Salmotruttasubsp.trutta	Sea trout
species	*Atherinaboyeri* Risso, 1810	Sand smelt
species	*Chelonauratus* (Risso, 1810)	Golden grey mullet
species	*Chelonlabrosus* (Risso, 1827)	Thicklip grey mullet
species	*Chelonramada* (Risso, 1827)	Thinlip grey mullet
species	*Mugilcephalus* Linnaeus, 1758	Flathead grey mullet
species	*Gasterosteusaculeatus* Linnaeus, 1758	Three-spined stickleback
species	*Dicentrarchuslabrax* (Linnaeus, 1758)	European seabass
species	*Platichthysflesus* (Linnaeus, 1758)	European flounder
species	*Lepomisgibbosus* (Linnaeus, 1758)	Pumpkinseed
species	*Micropterussalmoides* (Lacepède, 1802)	Largemouth bass
species	*Tincatinca* (Linnaeus, 1758)	Tench
species	*Gobiolozanoi* Doadrio & Madeira, 2004	Iberian gudgeon
species	*Carassiusauratus* (Linnaeus, 1758)	Goldfish
species	*Cyprinuscarpio* Linnaeus, 1758	Common carp

## Temporal coverage

**Data range:** 2010-1-05 – 2019-11-12.

### Notes

Sampling was performed on average every 6 days (5.7 ± 3.6) throught the study period, from January 2010 to November 2019. The interval between events was not constant and gaps of some days occurred, averaging 1.9 ± 3.5 (mean ± SD) days, with a minumum of 0 and a maximum of 34 days (Fig. 3).

## Usage licence

### Usage licence

Other

### IP rights notes

CC BY 4.0

## Data resources

### Data package title

Long-term monitoring of the fish community in the Minho Estuary (NW Iberian Peninsula)

### Resource link

https://doi.org/10.5281/zenodo.10321946

### Number of data sets

1

### Data set 1.

#### Data set name

Long-term monitoring of the fish community in the Minho Estuary (NW Iberian Peninsula)

#### Data format

TSV

#### Download URL

https://zenodo.org/records/10321946/files/DATASET_long-term-estuarine-fish_20231209_V05.tsv

#### Description

The dataset contains data from fyke nets deployed in the Minho Estuary (Portugal) from 2010 to 2019. The sampling frequency varied but, on average, data were collected using up to five different fyke nets. However, due to technical issues, the sampling pattern was not constant, with some fyke nets staying underwater for shorter or longer periods and occasionally having a variable number of fyke nets per event. The dataset includes 34 terms that follow Darwin Core standard (Darwin Core Maintenance Group 2021) whenever possible. These terms provide detailed information about the sampled organisms and their taxonomic classification. The dataset offers valuable insights into the biodiversity dynamics of the Minho Estuary ecosystem during the specific time period.

**Data set 1.** 

Column label	Column description
parentEventID	An identifier for the broader dwc:Event that groups this and potentially other dwc:Events.
eventID	An identifier for the set of information associated with an Event (something that occurs at a place and time). May be a global unique identifier or an identifier specific to the dataset.
eventDate	The date-time or interval during which an Event occurred. For occurrences, this is the date-time when the event was recorded. Not suitable for a time in a geological context.
year	The four-digit year in which the dwc:Event occurred, according to the Common Era Calendar.
startDayOfYear	The earliest integer day of the year on which the dwc:Event occurred (1 for January 1, 365 for December 31, except in a leap year, in which case it is 366).
endDayOfYear	The latest integer day of the year on which the dwc:Event occurred (1 for January 1, 365 for December 31, except in a leap year, in which case it is 366).
country	The name of the country or major administrative unit in which the dcterms:Location occurs.
countryCode	The standard code for the country in which the dcterms:Location occurs.
geodeticDatum	The ellipsoid, geodetic datum or spatial reference system (SRS) upon which the geographic coordinates given in dwc:decimalLatitude and dwc:decimalLongitude are based.
decimalLatitude	The geographic latitude (in decimal degrees, using the spatial reference system given in geodeticDatum) of the geographic centre of a Location. Positive values are north of the Equator, negative values are south of it. Legal values lie between -90 and 90, inclusive.
decimalLongitude	The geographic longitude (in decimal degrees, using the spatial reference system given in geodeticDatum) of the geographic centre of a Location. Positive values are east of the Greenwich Meridian, negative values are west of it. Legal values lie between -180 and 180, inclusive.
coordinateUncertaintyInMeters	The horizontal distance (in meters) from the given dwc:decimalLatitude and dwc:decimalLongitude describing the smallest circle containing the whole of the dcterms:Location. Leave the value empty if the uncertainty is unknown, cannot be estimated or is not applicable (because there are no coordinates).
DEIMS.iD	Unique alpha-numeric identifier of the site. The DEIMS-ID is automatically generated by DEIMS-SDR and adds the deims.org url as a prefix.
habitat	A category or description of the habitat in which the Event occurred.
basisOfRecord	The specific nature of the data record.
samplingProtocol	The names of, references to, or descriptions of the methods or protocols used during an Event.
sampleSizeValue	A numeric value for a measurement of the size (time duration, length, area or volume) of a sample in a sampling event.
sampleSizeUnit	The unit of measurement of the size (time duration, length, area or volume) of a sample in a sampling event.
samplingEffort	The amount of effort expended during a dwc:Event.
occurrenceStatus	A statement about the presence or absence of a dwc:Taxon at a dcterms:Location.
occurrenceID	An identifier for the dwc:Occurrence (as opposed to a particular digital record of the dwc:Occurrence). In the absence of a persistent global unique identifier, construct one from a combination of identifiers in the record that will most closely make the dwc:occurrenceID globally unique.
organismQuantity	A number or enumeration value for the quantity of organisms.
organismQuantityType	The type of quantification system used for the quantity of organisms.
degreeOfEstablishment	The degree to which an Organism survives, reproduces and expands its range at the given place and time.
vernacularName	A common or vernacular name.
scientificName	The full scientific name, with authorship and date information, if known. When forming part of an Identification, this should be the name in lowest level taxonomic rank that can be determined. This term should not contain identification qualifications, which should instead be supplied in the IdentificationQualifier term.
acceptedNameUsageID	An identifier for the name usage (documented meaning of the name according to a source) of the currently valid (zoological) or accepted (botanical) taxon.
taxonRank	The taxonomic rank of the most specific name in the scientificName.
kingdom	The full scientific name of the kingdom in which the taxon is classified.
phylum	The full scientific name of the phylum or division in which the taxon is classified.
order	The full scientific name of the order in which the taxon is classified.
family	The full scientific name of the family in which the taxon is classified.
genus	The full scientific name of the genus in which the taxon is classified.
scientificNameAuthorship	The authorship information for the scientificName formatted according to the conventions of the applicable nomenclaturalCode.

## Additional information

All sampling procedures were conducted in accordance with the European Directive 2010/63/EU on the protection of animals used for scientific purposes and its transposition into Portuguese law, "Decreto Lei" 113/2013.
